# Patient-centric knowledge graphs: a survey of current methods, challenges, and applications

**DOI:** 10.3389/frai.2024.1388479

**Published:** 2024-10-23

**Authors:** Hassan S. Al Khatib, Subash Neupane, Harish Kumar Manchukonda, Noorbakhsh Amiri Golilarz, Sudip Mittal, Amin Amirlatifi, Shahram Rahimi

**Affiliations:** Department of Computer Science and Engineering, Mississippi State University, Starkville, MS, United States

**Keywords:** knowledge graph, patient-centric, personalized healthcare, natural language processing, generative AI

## Abstract

Patient-Centric Knowledge Graphs (PCKGs) represent an important shift in healthcare that focuses on individualized patient care by mapping the patient’s health information holistically and multi-dimensionally. PCKGs integrate various types of health data to provide healthcare professionals with a comprehensive understanding of a patient’s health, enabling more personalized and effective care. This literature review explores the methodologies, challenges, and opportunities associated with PCKGs, focusing on their role in integrating disparate healthcare data and enhancing patient care through a unified health perspective. In addition, this review also discusses the complexities of PCKG development, including ontology design, data integration techniques, knowledge extraction, and structured representation of knowledge. It highlights advanced techniques such as reasoning, semantic search, and inference mechanisms essential in constructing and evaluating PCKGs for actionable healthcare insights. We further explore the practical applications of PCKGs in personalized medicine, emphasizing their significance in improving disease prediction and formulating effective treatment plans. Overall, this review provides a foundational perspective on the current state-of-the-art and best practices of PCKGs, guiding future research and applications in this dynamic field.

## 1 Introduction

The healthcare industry has experienced a significant shift, transitioning from traditional, provider-centric models toward patient-centered care. This shift highlights the critical role of engaging patients as active participants in their healthcare journeys. At the center of this transformation are PCKGs, which significantly advance personalized, data-driven care. PCKGs facilitate the integration of diverse data types, including medical history, genetics, lifestyle choices, and real-time data from health technology devices, fostering a comprehensive view of patient health essential for customizing treatments to individual needs (Mesko, 2022; Blobel, 2007).

The evolution of Knowledge Graphs (KG) in healthcare into sophisticated networks reflects an increasing acknowledgment of the complexity of human health and the insufficiency of siloed data systems in addressing multifaceted health issues. This evolution is propelled by the necessity for a holistic understanding of patient health, enabling personalized care and applying advanced data analytics to enhance healthcare outcomes (Blobel, 2011; Shirai et al., 2021). PCKGs stand at the forefront of healthcare innovation, signifying a crucial step toward integrated, patient-focused knowledge networks. This shift from fragmented data systems to cohesive KGs enables healthcare providers to employ machine learning and analytical technologies to help improve precision medicine, diagnostic accuracy, and treatment efficacy. Such a transition represents a technological leap that aligns with the broader objectives of healthcare reform aimed at delivering more personalized, efficient, and patient-centered care (Albannai et al., 2019; Almunawar and Anshari, 2012). However, incorporating KGs into healthcare presents technical, methodological, and ethical challenges, including data interoperability, privacy concerns, and the complexities of modeling diverse health outcomes. These hurdles pose significant barriers to the widespread adoption of PCKGs. Yet, the potential of these systems to revolutionize healthcare by offering a nuanced and comprehensive understanding of patient health is unquestionable (MacLean, 2021; Alagar et al., 2017).

PCKGs’ primary aim is to enhance the quality of patient care, improve treatment outcomes, and increase the efficiency of healthcare delivery. By integrating disparate data sources and utilizing advanced analytical models, PCKGs promise to deliver personalized, efficient, and effective healthcare services tailored to each patient’s unique needs. This goal emphasizes the shift toward a healthcare system that values and prioritizes individual patient experiences and needs, marking the beginning of a new era of patient-centric, data-driven care (Harper and Honour, 2015; Goniewicz et al., 2021). Despite the inherent challenges in integrating KGs into healthcare, the critical need for the advanced application of PCKGs to achieve personalized care and enhance healthcare delivery systems is undeniable. As the healthcare landscape continues to evolve, PCKGs exemplify the industry’s commitment to leveraging technology to meet patients’ complex and varied needs, thus marking a significant milestone in the journey toward more personalized and effective healthcare solutions.

The motivation for this paper stems from the growing need to consolidate disparate healthcare data into a unified, holistic view for improved patient care. The key contributions of this survey paper are:

- A foundational explanation of knowledge graphs serves as the theoretical basis for the remainder of the paper.
- Presentation of survey findings and introduction of a taxonomy developed for PCKGs.
- An in-depth review of methodologies designed explicitly for PCKGs, shedding light on the most effective techniques currently employed.
- Exploration of real-world applications and use cases that have successfully implemented PCKG methodologies, providing evidence of their utility.

The rest of the paper is organized as follows: Section 2 explains the principles of knowledge graphs, providing a foundation for the following discussions. Section 3 presents the findings from our survey and introduces the taxonomy we have developed. Moving on to Section 4, we review methodologies explicitly designed for PCKGs. Section 5 explores real-world applications and examples that benefit from these methodologies. Section 6 highlights research challenges and provides targeted recommendations for future scholars in this field. Finally, in Section 7, we summarize the key findings of this paper and outline directions for work in PCKGs.

## 2 Background

The development of knowledge representation has a rich history in the realms of logic and AI. The notion of graphical knowledge representation can be traced back to 1956 when Richens (1956) introduced the concept of semantic nets. Similarly, symbolic logic knowledge finds its roots in the General Problem Solver (Newell et al., 1959) of 1959. Initially, knowledge bases were employed in knowledge-based systems for reasoning and problem- solving. Notably, MYCIN (Shortliffe, 2012), an expert system renowned for medical diagnosis, utilized a knowledge base containing approximately 600 rules. However, it was in 2012 that the concept of Knowledge Graph (KG) gained immense popularity, thanks to Google’s search engine and its introduction of the Knowledge Vault framework (Dong et al., 2014). This framework aimed to construct large-scale KGs through knowledge fusion. There is currently no consensus on the definition of the term, with several authors proposing different definitions. Table 1 illustrates some of these definitions presently available in the literature.

**Table 1:** Various definitions of KGs are in the available literature.

Source	Definition
Paulheim (2017)	A knowledge graph (i) mainly describes real world entities and their interrelations, organized in a graph, (ii) defines possible classes and relations of entities in a schema, (iii) allows for potentially interrelating arbitrary entities with each other and (iv) covers various topical domains
Ehrlinger and Wolfram (2016)	A knowledge graph acquires and integrates information into an ontology and applies a reasoner to derive new knowledge
Wang et al. (2017)	A knowledge graph is a multi-relational graph composed of entities and relations which are regarded as nodes and different types of edges, respectively
Smirnova et al. (2018)	A Knowledge Graph, also known as a Knowledge Base, is a directed graph formed by triples that connect nodes (subjects) to other nodes (objects) through properties (predicates), where these connections represent semantic relationships and the graph’s structure illustrates the subjects, objects, and their interrelations
Duan et al. (2017)	A knowledge graph organizes items, entities, and users as nodes interconnected by edges, providing rich semantics and comprehensive information in a structure akin to natural language

**Figure 1:**
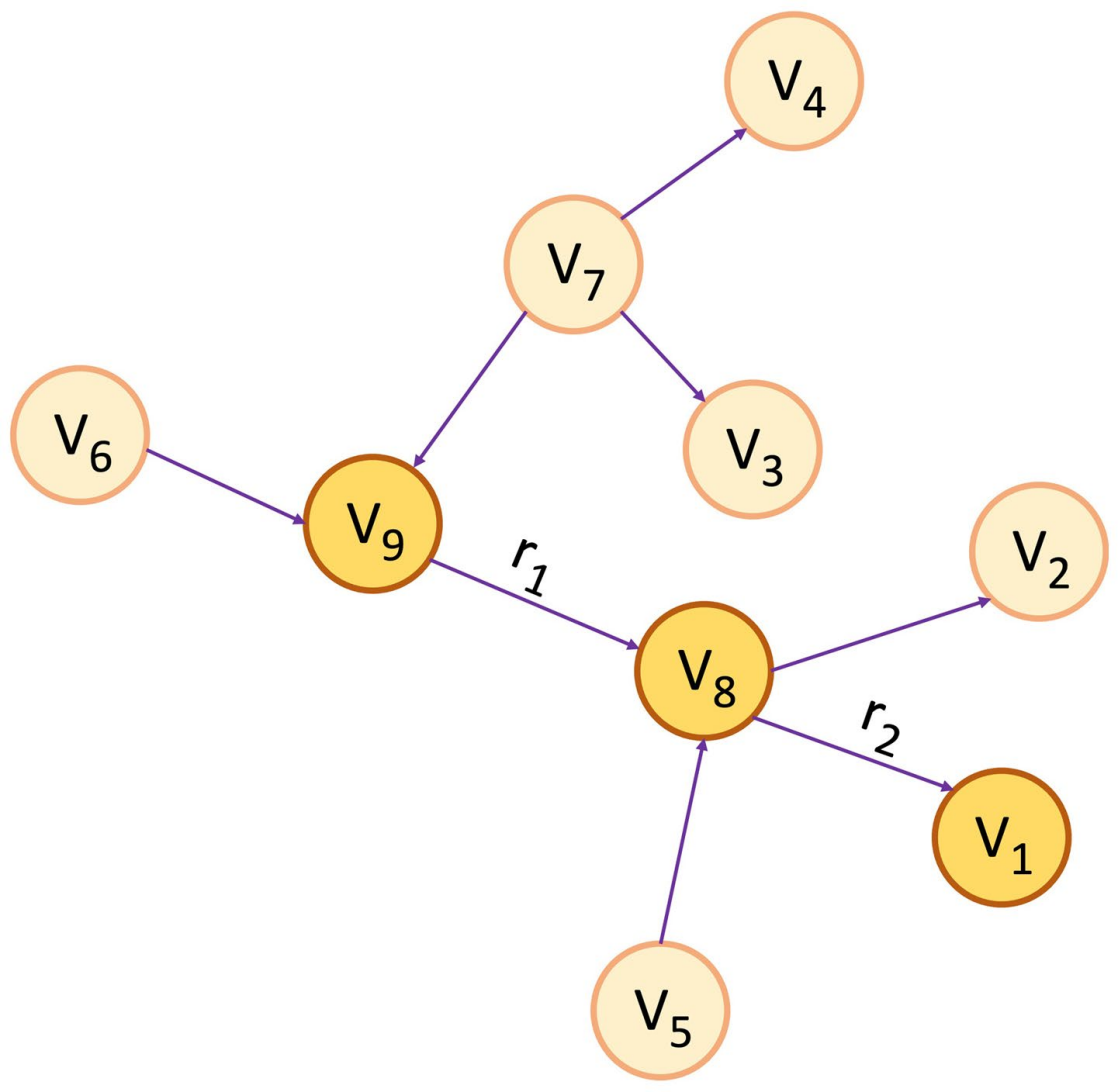
An example of a knowledge graph, where the triplet (*v*_9_, *r*_1_, *v*_8_) serves as an illustration of the link between entities *v*_9_ and *v*_8_ through the relationship *r*_1_ and (*v*_8_, *r*_2_, *v*_1_) through *r*_2_ for the relationship between *v*_8_ and *v*_1_.

**Figure 2:**
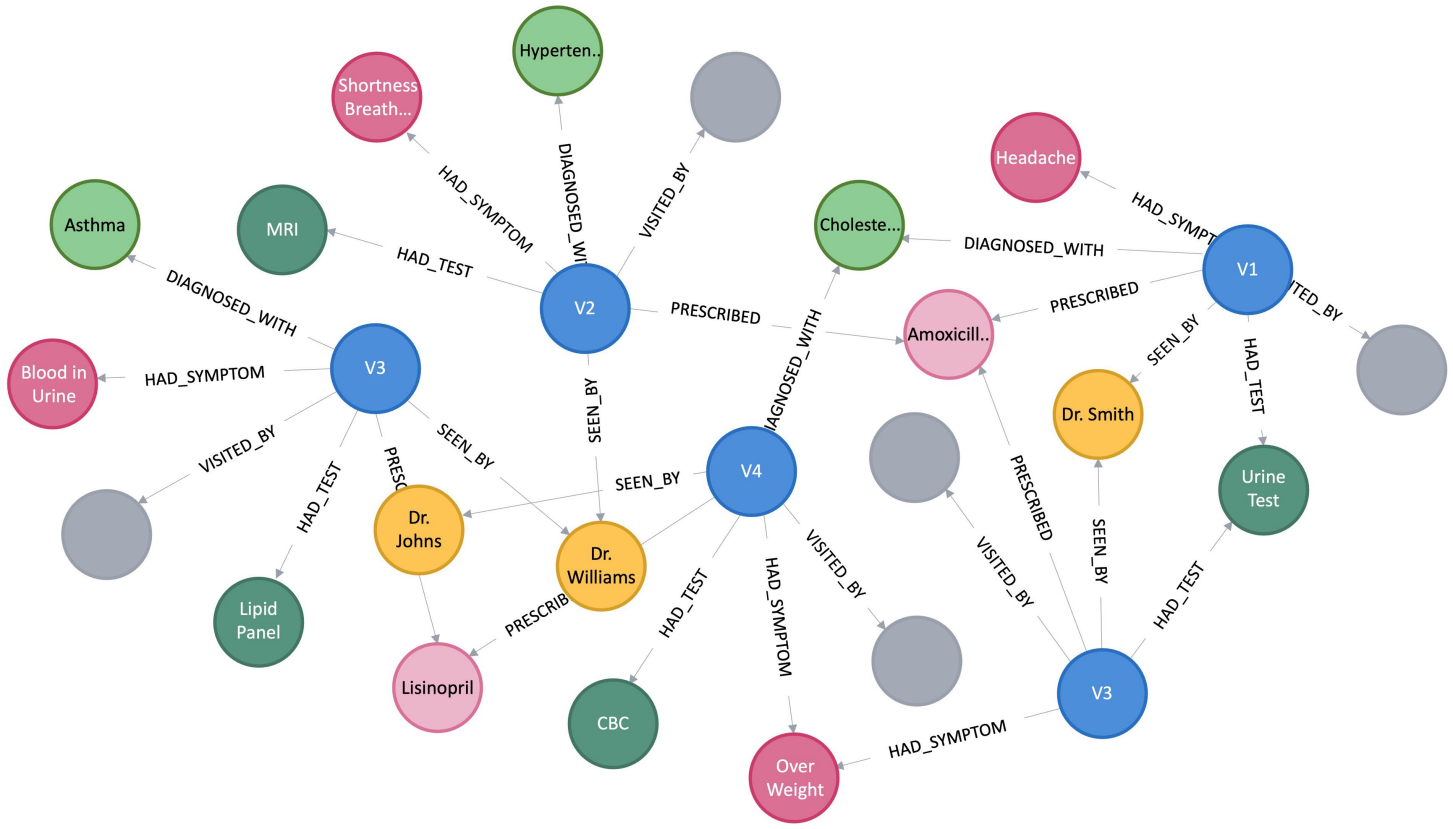
Illustration of patient’s clinical visits knowledge graph.

A KG assumes the form of a directed graph (*G*), characterized by vertices and edges, where *G* = (*V, E*). Vertices (*V*) indicate an entity, and Edge (*E*) between the two vertices convey the semantic relationship between two entities (Bordes et al., 2011). Within the graph, vertices, also referred to as entities or nodes, are linked through relationships represented as edges, and facts are depicted using RDF (Cyganiak et al., 2014) triples such as (subject, predicate, object) or (head, relation, tail), denoted by *< h, r, t >* (Abu-Salih, 2021). Figure 1 depicts a simple KG with vertex *v*_9_ and *v*_8_ linked by the relation *r*_1_, which goes from *v*_9_ to *v*_8_, making a triplet (*v*_9_, *r*_1_, *v*_8_). In this scenario, *v*_9_ represents the head, and *v*_8_ represents the tail. In the context of PCKGs, nodes typically represent entities like patients, drugs, diseases, or genes, whereas edges denote relationships or associations between these entities (see Figure 2).

Over the past few years, especially within the domains of health and biomedicine, various forms of KGs have been proposed, often stemming from literature or Electronic Health Records (EHR). However, they are typically not individual-centered. Recent work on disease-centric knowledge graphs is available in Chandak et al. (2023) and Lin et al. (2023). These KGs facilitate clinicians’ ability to study the relationships between diseases to answer practical clinical problems. Nevertheless, because relationships between disease concepts are frequently distributed across numerous datasets, integrating multimodal data sources is critical for developing comprehensive disease knowledge graphs (Chandak et al., 2023; Lin et al., 2023). On the other hand, their robustness of knowledge in terms of diseases and symptoms must be evaluated to assess their accuracy and quality (Chen et al., 2019).

In hospitals, patient information collected during visits and details of test results, clinical notes, symptoms, diagnoses, and drugs is frequently maintained as an EHR. These records include both unstructured data in the form of free notes, such as progress notes, admission notes, discharge summaries, medical histories, procedures notes, etc., and structured data, such as medical codes, medications, administrative data, vital signs, and laboratory test report data (Sarwar et al., 2022). Numerous KGs have been introduced in the literature, utilizing EHRs as their foundation. As an illustration, Finlayson et al. (2014) constructed a graph including diseases, drugs, procedures, and devices. They leveraged 1 million clinical concepts from 20 million clinical notes to do so. Similarly, another study in Rotmensch et al. (2017) formulated a KG covering 156 diseases and 491 symptoms based on the insights drawn from a dataset of 273,174 patient visits to the emergency department. Although some efforts have been made to incorporate patient-specific information into these graphs, they are not yet patient-centric.

PCKGs can be linked to Personal Knowledge Graphs (PKG), an emerging concept in this field. Although PKGs are relatively new, some notable contributions have been made in this area. One of the pioneering papers that formally introduces the concept of PKG is authored by Balog and Kenter (2019). They define PKG *“to be a source of structured knowledge about entities and the relation between them, where the entities and the relations between them are of personal, rather than general, importance. The graph has a particular “spiderweb” layout, where every node in the graph is connected to one central node: the user.”* Within the scope of this definition, the PKG only incorporates knowledge pertinent to the specific user.

The emergence of KGs has brought about a substantial transformation in the healthcare domain, providing numerous advantages to this industry. Integrating disparate health data to provide a comprehensive and holistic view of patient health is a significant application of KGs. In the era of precision medicine, wherein therapeutic strategies are customized to suit individual patients’ distinctive genetic composition and lifestyle variables, KGs assume a crucial function. Integrating various data types, such as genetic profiles, medical history, lifestyle behaviors, and information from health technology devices, is facilitated to enhance efficiency. Implementing this all-encompassing methodology enables more accurate diagnoses, treatment strategies, and potentially enhanced health results (Johson et al., 2021).

Moreover, KGs are a formidable instrument for deciphering intricate biomedical data, resulting in amplified research findings (Bauer-Mehren et al., 2010). They provide a systematic depiction of connections among different entities, such as genes, diseases, drugs, and pathways, which can be utilized to formulate novel hypotheses and uncover concealed patterns. For example, KGs can elucidate the intricate relationship between a genetic mutation and its association with a specific disease or unveil the influence of environmental factors in the progression of said disease. As mentioned earlier, the discoveries possess the potential to significantly contribute to the development of groundbreaking therapeutic approaches and expand the horizons of biomedical investigation (Yuan et al., 2011).

KGs play a crucial role in developing effective decision-support systems in the clinical domain. By effectively amalgamating and analyzing individualized patient data, KGs can assist medical practitioners in formulating accurate diagnostic determinations, devising optimal treatment strategies, and forecasting patient prognoses. Consequently, the implementation of KGs can effectively mitigate the occurrence of medical errors (Raghupathi and Raghupathi, 2014).

KGs have found significant applications across various sectors beyond healthcare. For example, in e-commerce, KGs are extensively used to enhance user experience through personalized recommendations, search result ranking, and semantic search. Prominent e-commerce platforms, such as Walmart, Amazon and eBay, have been utilizing KGs to link items to their respective attributes, thereby providing more relevant product suggestions to customers (Xu et al., 2020). Moreover, KGs are increasingly used in Natural Language Processing (NLP), where they can improve the performance of machine translation, question-answering, and text summarization systems (Li et al., 2022). KGs also have substantial potential in cybersecurity, as they can aid in mapping relationships between cyber threats, vulnerabilities, and affected systems, facilitating better threat prediction and prevention (Liu et al., 2022). Social networking platforms like Meta and LinkedIn also leverage KGs to understand user relationships and interests, enhancing content recommendation and advertising targeting strategies (He et al., 2020). Hence, the applications of KGs are diverse and significantly impact various domains, reinforcing their relevance and potential.

## 3 Developed taxonomy of patient-centric knowledge graph

Having introduced the concept of KG in the previous section, we now explore the survey’s core. This section will examine the various approaches, categorizations, and details influencing PCKGs. PCKGs are characterized by a variety of methodologies and studies. To navigate this diverse landscape, we need a well-structured framework. In this context, a taxonomy is a guide that enables readers to comprehend and classify the complexities involved in PCKG construction, evaluation, processing, and applications.

**Figure 3:**
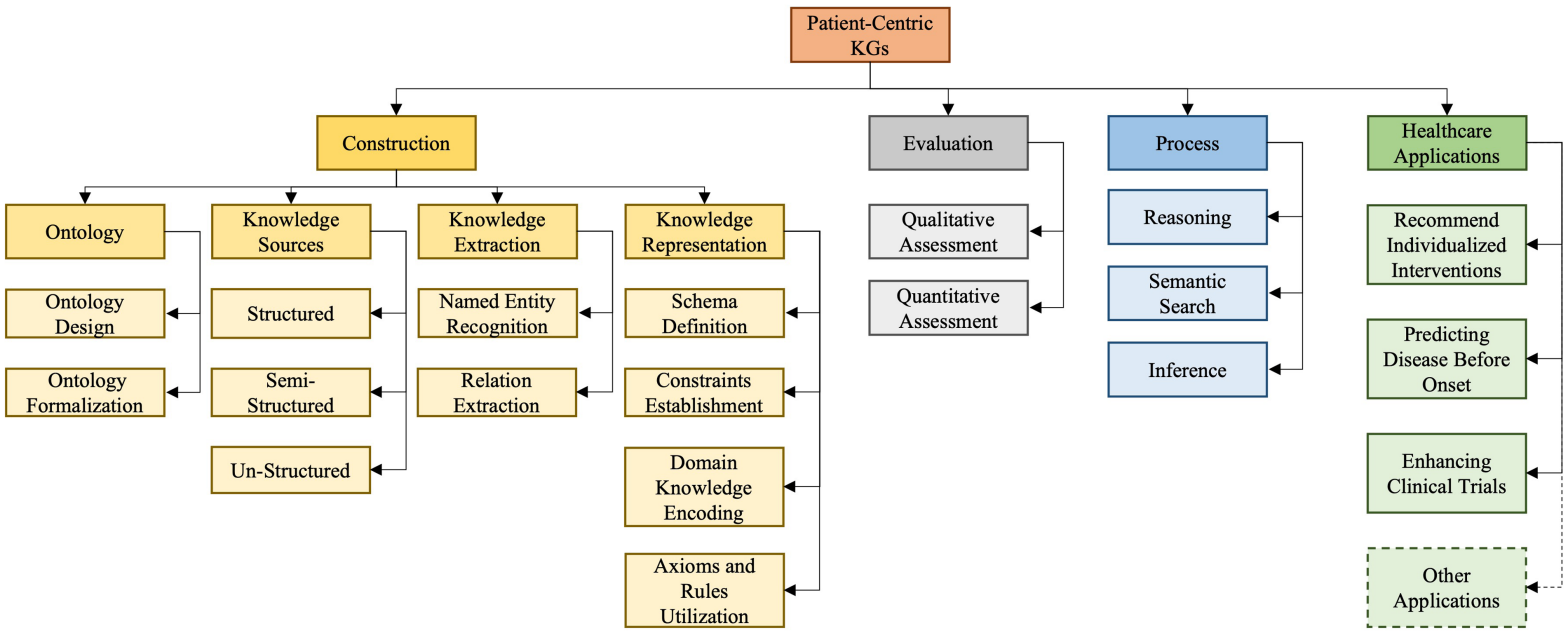
Proposed taxonomy of patient-centric knowledge graph.

As depicted in Figure 3 our developed taxonomy is derived from an exhaustive survey and analysis of existing literature and practices (Eggert and Alberts, 2020). Based on our findings, we classify PCKG taxonomy into four major categories, including *construction, evaluation, process, and applications*. Given the heterogeneity of studies in the domain, it provides a structured representation and insight into the why and how of each categorization. The taxonomy presented in this paper outlines essential relationships and highlights the critical aspects of each of these categories. Its uniqueness lies in its exhaustive nature, informed by the foundational literature and cutting-edge research. In the following paragraphs, we delve into each category and subcategory.

The first category pertains to the *construction* of a PCKG and is further divided into four sub-categories: *ontology, knowledge sources, knowledge extraction, and knowledge representation.*

The initial step, *Ontology*, revolves around designing structured frameworks custom-tailored to meet patient-centered needs, ensuring that the graph accurately captures the essence of patient data while adhering to industry standards and best practices. It is crucial to articulate distinctions among closely related terms in this context: a Knowledge Graph (KG), which represents interconnected real-world facts; a Knowledge Base (KB), which stores this structured information; and an ontology, which provides a structured framework that illustrates and organizes concepts, ensuring consistent understanding and interpretation of the data within the KG and KB. Second, considering the diverse nature of healthcare data, our *Knowledge Sources* segment highlights the differences and integration methods for three primary sources of data: structured, semi-structured, and unstructured data. Third, the *Knowledge Extraction* phase employs techniques like Named Entity Recognition (NER) to pinpoint essential entities such as drugs or symptoms (Keretna et al., 2015). The process is complemented by Relationship Extraction (RE), which accurately maps the relationships among these entities, giving the graph its characteristic interconnected structure (Wang et al., 2021). The last sub-category in construction is *Knowledge Representation*, which focuses on detailing the methodological approach of encapsulating explicit and implicit knowledge in the PCKG. This involves defining the schema for entities and relationships, establishing constraints, and encoding domain knowledge through axioms and rules, thus constructing a robust framework that effectively translates raw data into a comprehensible and interconnected knowledge graph that accurately mirrors the complexity and depth of patient-centric data.

The second category involves the evaluation of the PCKG, emphasizing the need to assess the efficacy and accuracy of PCKGs post-construction. This assessment can be facilitated by two principal methodologies: *qualitative* and *quantitative*. The former involves a thorough examination of the graph to ensure that it adheres to clinical best practices and contains relevant and correct medical relationships. The latter uses statistical and computational methodologies to evaluate the graph’s performance metrics, including accuracy, recall, and precision. It also offers a numerical assessment of its ability to represent and infer knowledge accurately and effectively. Once the PCKG has been constructed and evaluated, the next step is its *utilization (processing PCKG)* to derive actionable insights. To attain this objective, various approaches are crucial, including using methods such as *reasoning, semantic search, and inference*. *Reasoning* enables the PCKG to logically extract new information and knowledge from the existing knowledge within the graph, providing a basis for making intelligent decisions and predictions about healthcare outcomes and strategies (Rajabi and Kafaie, 2022). In contrast, *Semantic Search* allows structured searches to retrieve precise information from the graph (Lytras et al., 2009). *Inference*, on the other hand, uses the inherent structure of the graph to derive new insights and conclusions (Wang et al., 2018).

The final category of the our taxonomy relates to the *application* of PCKG in the healthcare domain. We explore four primary healthcare use cases, encompassing *recommending individualized interventions, clinical trials, predicting disease before onset, and others.* The first personalizes patient care treatments based on insights from the graph. The second application aims to enhance the efficiency of *Clinical Trials*, aiding in developing trial designs or optimizing patient selection. On the other hand, a particularly innovative application is *Predicting Disease Before Onset*, where the complex patterns and relationships mapped in the graph can be harnessed to detect potential health issues proactively. Although these are highlighted applications, the potential of PCKGs extends even further, covering areas such as *drug discovery, personalized medication regimens,* and *preventive health strategies*, to name a few.

In the following sections, we will examine each component of our taxonomy in more detail to better understand its significance and provide an overview of the suggested implementation steps.

## 4 Construction, evaluation and processing of PCKGS

This section explores the complex processes and methodologies in developing, assessing, and utilizing PCKG. This comprehensive exploration is structured into four critical categories, as illustrated in Figure 3. The first category, *construction* of PCKG, explains the complex process of building PCKG, highlighting the significance of ontology, various data sources, knowledge extraction techniques, and knowledge representation. Next the *evaluation* covers qualitative and quantitative methods for assessing the efficacy and accuracy of these graphs. Similarly, we discuss advanced techniques like *reasoning, semantic search*, and *inference*, which are essential to harnessing the full potential of PCKGs to derive actionable insights and inform healthcare decisions.

### 4.1 PCKG construction

The process of creating PCKGs involves four steps. To begin with, the *ontology* phase involves developing structured frameworks based on patient data, which ensure accuracy and adherence to standards. *Knowledge sources* are the second step, which addresses the collection and integration of diverse types of healthcare data, including structured, semi-structured, and unstructured data. During the third step, *knowledge extraction*, key data entities and their interconnections are mapped using named entity recognition and relationship extraction techniques. Lastly, *knowledge representation* involves defining a schema for entities and relationships, setting constraints, and encoding domain knowledge to transform raw data into a coherent, interconnected graph reflecting patient data complexity. In the following subsections, we will explore these steps in detail.

#### 4.1.1 Ontology

Ontologies provide a structured framework for representing knowledge, integrating diverse data sources, and facilitating sophisticated access to health information—critical components in patient-centered healthcare (Puustjarvi and Puustjarvi, 2010). Designing and formalizing ontologies involves creating a comprehensive schema that unifies information from various *structured* and *unstructured* data sources (Iannacone et al., 2015). Unlike knowledge bases, which are collections of domain-specific knowledge often built upon ontologies or schemas, which primarily organize data as a blueprint for databases, ontologies formally represent concepts within a domain and the relationships between them, offering a deeper contextual understanding. In healthcare, the application of ontologies has been well-documented. For instance, Sun et al. (2014) introduced an ontology-enabled healthcare service model that supports joint referral decisions between patients and general practitioners, enhancing patient-centered care.

One practical implementation of ontology principles is the Fast Healthcare Interoperability Resources (FHIR) standard. FHIR plays a crucial role in structuring patient-centric data, enabling the representation of complex healthcare information—such as patient records, clinical observations, and care plans—in a machine-readable and interoperable format (Rishi Kanth Saripalle, 2019). FHIR uses a resource-based model with RESTful APIs, which supports formats like JSON, XML, and RDF, and can be integrated into graph-based models, including PCKGs, to enhance data relationships. By integrating FHIR-based models into PCKGs, we can achieve enhanced interoperability and comprehensive patient data mapping. This ensures that PCKGs accurately reflect the multifaceted nature of patient health information, supporting standardized healthcare data exchange and facilitating more effective integration across diverse healthcare systems (Maxhelaku, 2019).

##### 4.1.1.1 Design

**Figure 4:**
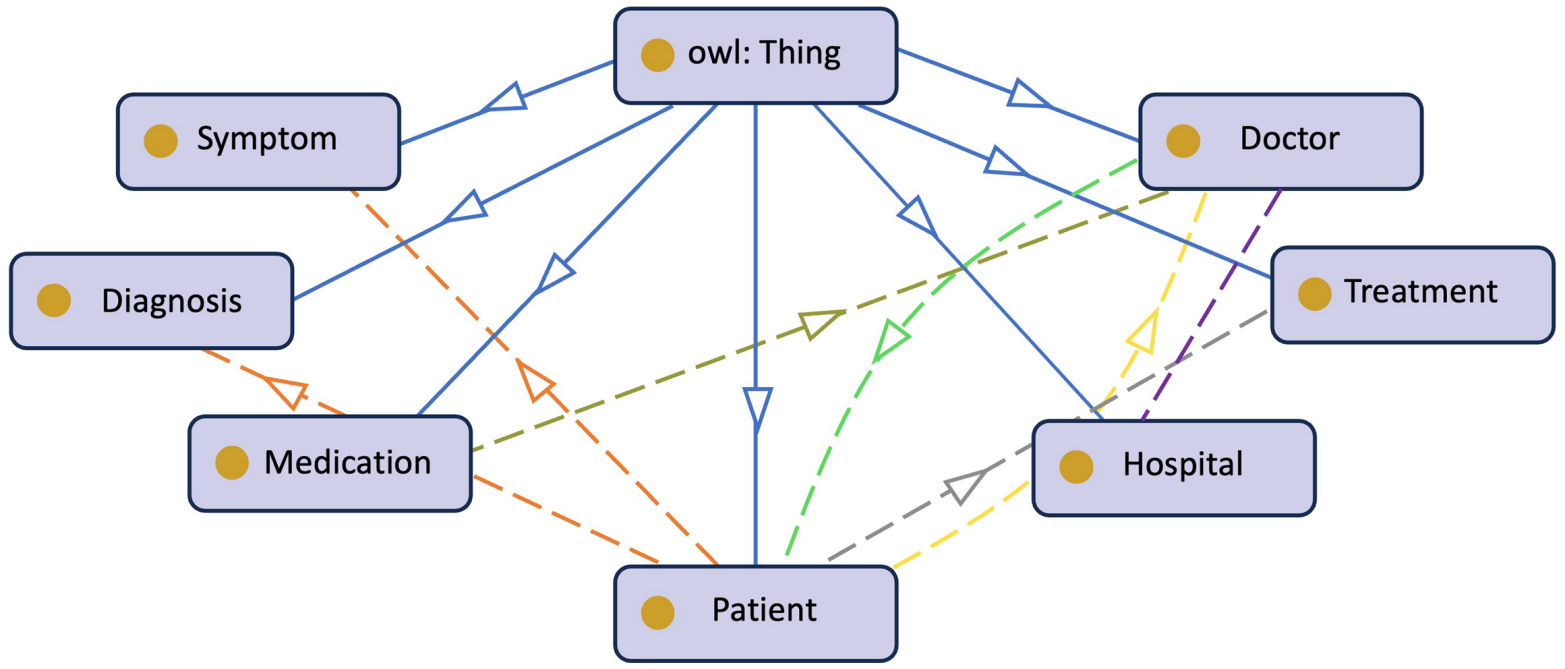
Illustration of a basic patient’s ontology.

The growing digitization of healthcare data and the proliferation of medical information need effective and structured methods of representing and managing patient-centric knowledge. Ontologies, formal representations of knowledge, play a critical role in healthcare systems’ interoperability, data integration, and decision support (Taye, 2010). In the context of PCKGs, ontology design is a fundamental process that involves defining and modeling the important entities, connections, and features related to patient health and treatment. Figure 4 provides an example of a patient’s ontology. Authors in Noy and McGuinness (2023) argue that the rationale behind ontology development is to aid in a shared understanding of information structure among people or software agents, facilitate the reuse of domain knowledge, make domain assumptions explicit, separate domain knowledge from operational knowledge, and analyze domain knowledge. However, there is no standard methodology (Kapoor and Sharma, 2010) or correct way of formulating ontology (Noy and McGuinness, 2023).

The initial stage in ontology design is to establish the scope and domain of the PCKG. The scope of the ontology specifies the breadth of medical knowledge included. In contrast, the domain defines the precise areas of concentration, such as general medical information, specific diseases, or healthcare procedures. Identifying the domain aids in the selection of acceptable current ontologies such as SNOMED-CT (El-Sappagh et al., 2018), RxNorm (Hanna et al., 2013), and LOINC (Uchegbu and Jing, 2017) are healthcare standards that can be utilized to maintain interoperability and consistency. While current ontologies and standards may provide a solid foundation, some patient-centric concepts may not be fully covered. In such circumstances, additional classes and features must be added to cater to specific requirements. This requires collaboration between domain experts and ontology engineers to guarantee that the ontological representations are consistent with healthcare practices and guidelines.

The second step is to define the core entities. Entities such as *Patient*, *Disease*, *Medication*, *Symptom*, *Medical Procedure*, *Healthcare Provider*, etc. are typically included. These entities serve as the building blocks for constructing the KG and are crucial for capturing patient-related information.

The next step is to extract or specify the relationships between entities. This step is essential for capturing the complex interactions within patient-centric healthcare. Relationships, such as *Diagnosis, Treats, Has Symptom*, and *Undergoes,* enable meaningful associations and support inferencing capabilities. During this stage, the ontology developer should also carefully consider the attributes and properties of the entities. For example, attributes of a *patient* could be *age*, *gender*, *sex*, *demography*, etc. These attributes can influence the diagnosis and treatment decisions (Bravo, 2019).

##### 4.1.1.2 Formalization

Formalizing the ontology focuses on structuring and representing patient data and medical knowledge in a way that is understandable and accessible to healthcare systems. Puustjarvi and Puustjarvi (2010) leveraged the Resource Description Framework (RDF) and Web Ontology Language (OWL) to facilitate sophisticated access to health information, a cornerstone for effective patient care. This innovative approach underscores the evolving landscape of healthcare informatics, where the structuring and accessibility of patient data are critical. Similarly, Jiang et al. (2003) provided insight into the role of Formal Concept Analysis (FCA) in developing ontologies within clinical domains. FCA emerges as a key tool, offering linguistic and context- based knowledge. This knowledge is indispensable for clinical experts, aiding them in comprehending and applying ontology effectively in their practice. Integrating FCA into ontology development signifies a deeper understanding of clinical data and its nuances, enhancing the overall utility of these ontologies in real-world medical settings.

Further broadening this scope, Djedidi and Aufaure (2007) presented a methodology that underscores the integration of heterogeneous data sources with existing ontologies and standards. This approach is instrumental in constructing a medical domain ontology as a comprehensive, knowledge-centric decision support system. Such integration is crucial in ensuring the developed ontology is robust and aligns seamlessly with existing medical knowledge frameworks and data sources. Likewise, Mendes et al. (2013) contributed significantly to the Ontology for General Clinical Practice (OGCP) development. This ontology extends the Ontology for General Medical Science (OGMS) by incorporating the Clinical.

Patient Record (CPR) structure. This extension significantly enhances the representation and reasoning capabilities within clinical practice knowledge, especially in the context of natural language text. The OGCP stands as a testament to the evolving complexity and sophistication required in modern medical ontologies, catering to the nuanced needs of clinical practice.

The dynamic nature of medical knowledge and patient data necessitates methodologies for tracking and updating information. In this context, Ognyanov and Kiryakov (2002) proposed a formal model for tracking changes in RDF(s) repositories. This model is vital for maintaining the accuracy and relevance of the KG over time, addressing the ever-changing landscape of medical data and knowledge. Clarkson et al. (2018) introduced a user-centric methodology for the ontology population to address the user perspective. This approach aligns user concepts with target ontologies, proving an efficient method for building and maintaining ontologies across various domains. The user-centric approach ensures that the developed ontologies are technically sound and resonate with the needs and understandings of the end-users, be they patients or healthcare professionals.

Ferilli (2021) proposed an intermediate format that can be easily mapped onto formal ontology for complex reasoning and a graph database for efficient data handling to bridge the gap between formal ontology and practical application. This innovation represents a significant stride in harmonizing the theoretical aspects of ontology with the practical demands of data management, ensuring that the developed ontologies are both conceptually sound and practically applicable.

Together, these diverse yet interconnected research efforts paint a comprehensive picture of ontology formalization’s current state and future potential in patient-centric healthcare. They highlight a collaborative and multi-faceted approach to developing technically robust ontologies that are deeply integrated with the practical realities of healthcare delivery.

#### 4.1.2 Knowledge sources

**Figure 5:**
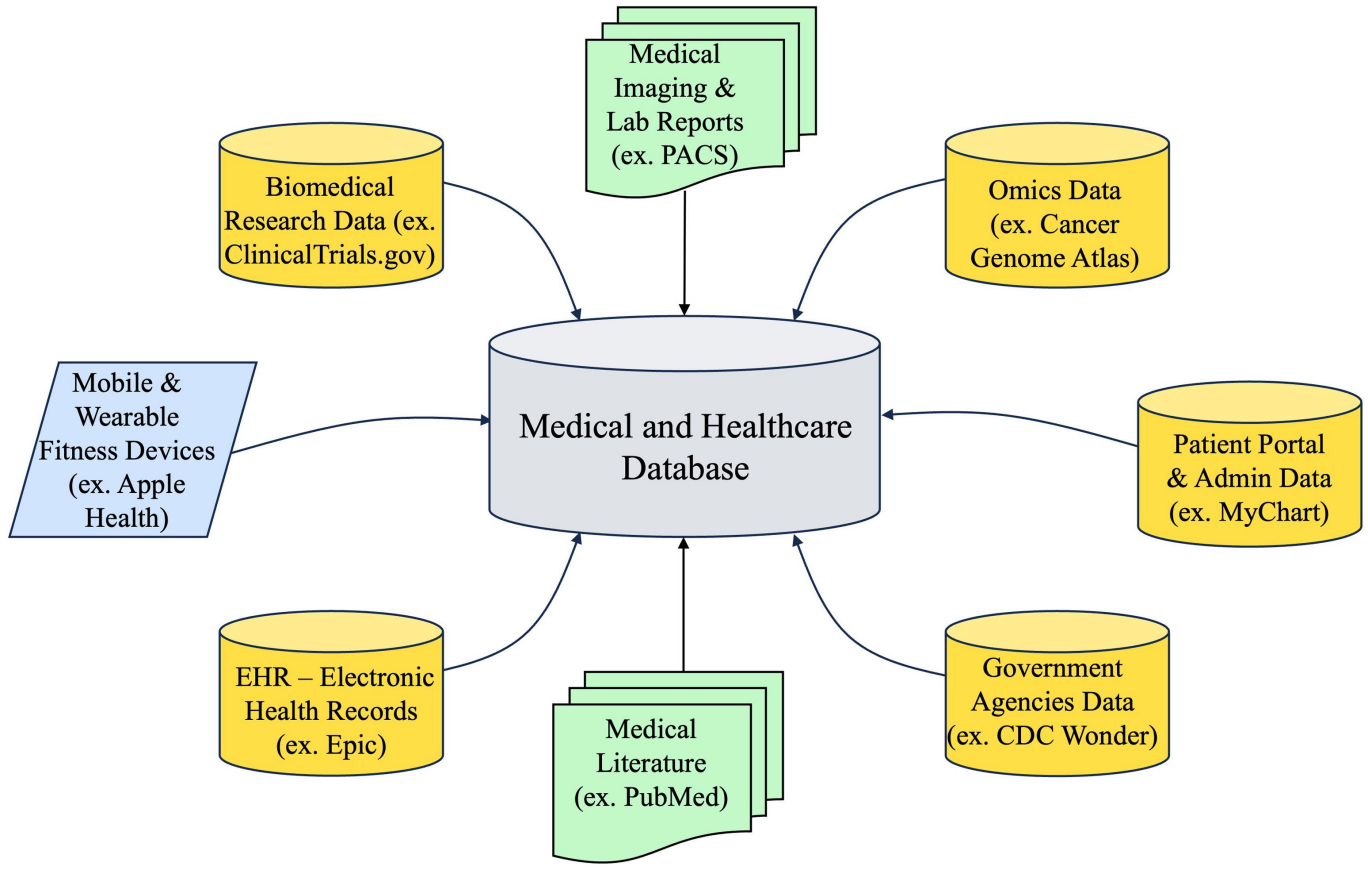
Diverse sources of medical and healthcare data with examples.

PCKGs are complex structures that integrate diverse data sources, as illustrated in Figure 5, to offer a comprehensive perspective on a patient’s medical past, present ailments, and prospective therapies. The extent of a PCKG’s depth is contingent upon the diversity and quality of the data sources that contribute to its composition. The sources can be classified into three main categories: *structured, semi-structured, and unstructured data*.

##### 4.1.2.1 Structured data

Structured data is organized and easily searchable in relational databases. It follows a specific schema or model, making it straightforward to query and analyze. Structured healthcare data, integral to PCKGs, is exemplified by *EHRs, lab results and diagnostics*, and *genomic and molecular data*. *EHRs*, as digital records of a patient’s medical history, encompass diagnoses, medications, treatment plans, and vital statistics, providing a highly structured backbone for PCKGs. Transitioning to *lab results and diagnostics*, this category includes structured, numerical, or categorical data from blood tests, imaging studies (like X-rays and MRIs), and other diagnostic tests, facilitating their integration into PCKGs. Lastly, *genomic and molecular data* derived from genetic tests offer structured insights into a patient’s disease predisposition, proving essential for advancing personalized medicine. Collectively, these structured data forms are fundamental in healthcare analytics and personalized treatment planning.

##### 4.1.2.2 Semi-structured data

Semi-structured data does not reside in a relational database but does have some organizational properties that make it easier to analyze. It often requires some level of transformation to be fully utilized. Semi- structured healthcare data encompass *clinical notes* and *patient-generated data*, each possessing unique characteristics that bridge the gap between structured and unstructured data. While fundamentally unstructured in text, *clinical notes and narratives* often adhere to templates or contain tags, rendering them semi-structured and providing contextual depth absent in purely structured datasets. Similarly, *patient- generated data*, sourced from wearables like fitness trackers and mobile health apps, presents structured data points (e.g., heart rate, steps) but lacks a consistent schema overall, contributing to the semi-structured nature of the data set. This combination of structured elements within an otherwise unstructured framework is valuable for comprehensive healthcare data analysis.

##### 4.1.2.3 Unstructured data

Unstructured data is information that does not have a pre-defined data model, making it challenging to analyze using conventional methods. Unstructured healthcare data, characterized by its non-standardized format, includes *patient surveys*, *interviews*, *medical literature*, and *research papers*, each offering unique challenges and insights for PCKGs. *Patient surveys and interviews*, typically in text or audio formats, yield qualitative insights into patients’ conditions but are unstructured, necessitating advanced analytics for effective integration into PCKGs. Similarly, *medical literature and research papers* are a rich source of valuable insights but remain unstructured; thus, they require NLP techniques to distill and extract relevant information for PCKG incorporation. With its inherent complexity, this unstructured data is crucial in enhancing the depth and breadth of healthcare analytics and patient care understanding.

By integrating these diverse data sources, PCKGs can offer a multi-dimensional view of a patient’s health, enabling more personalized and effective healthcare interventions.

#### 4.1.3 Knowledge extraction

Knowledge extraction plays a pivotal role in constructing PCKGs. This stage leverages techniques such as Named Entity Recognition (NER) and Relationship Extraction (RE) to distill valuable insights from unstructured data. While NER focuses on identifying and categorizing key entities in the data, RE takes a step further to determine the relationships between these entities. A structured and rich KG can be constructed through the harmonious interplay of NER and RE, paving the way for more personalized and efficient healthcare solutions.

##### 4.1.3.1 Named entity recognition

NER identifies and classifies entities such as diseases, symptoms, and medications from unstructured text. This process is fundamental in transforming raw data into structured, actionable information. Recent advancements in NER methodologies have significantly enhanced the development of KGs, particularly in the medical domain. For instance, Li et al. (2019) introduced the BiLSTM-Att-CRF model, which integrates an attention mechanism and part-of-speech features to improve clinical NER in Chinese electronic medical records. Building on this, Keretna et al. (2015) proposed a graph-based technique that enhances medical NER performance by up to 26% in unstructured medical text, showcasing the versatility of NER applications.

Furthering these developments, Wang and Haihong (2021) developed a bi-directional joint embedding model that combines encyclopedic knowledge with original text, thereby showing improved results in Chinese medical NER. Complementing this approach, Wang et al. (2018) demonstrated that incorporating dictionaries into deep neural networks effectively addresses the challenge of rare and unseen entities in clinical NER, thus broadening the scope of NER applications. In a similar vein, Guan and Tezuka (2022) highlighted the importance of entity linking and intent recognition in medical question-answering systems, significantly improving medical KG searches’ efficiency and accuracy. In a similar context, Zhou et al. (2021) developed a multi-task adversarial active learning model that enhances medical NER and normalization by considering task-specific features, further illustrating the dynamic evolution of NER methodologies.

Applying Large Language Models (LLMs) for NER is increasingly considered pivotal in healthcare- related KGs. Recent advancements in LLMs have demonstrated their effectiveness in improving NER tasks by leveraging contextual embeddings to enhance the accuracy of entity recognition, particularly in complex biomedical texts (Liu and Fang, 2023; Iscoe et al., 2023). For instance, fine-tuning LLMs on specific medical datasets has shown promising results in identifying entities related to patient care, thus enriching the KG with valuable insights that support personalized medicine (Hafsah et al., 2023). Furthermore, integrating ontological knowledge into LLM-driven NER processes can significantly enhance the interpretability and robustness of the extracted information, making it more applicable to clinical applications (Liu et al., 2022).

These diverse methodologies reflect the evolving landscape of NER in developing PCKGs. Integrating advanced machine learning techniques, attention mechanisms, LLMs, and domain-specific knowledge has led to more accurate and efficient entity recognition, which is crucial for effectively utilizing KGs in healthcare.

##### 4.1.3.2 Relation extraction

Like NER, RE is a fundamental process in transforming unstructured data into a structured form, enabling more effective healthcare and medical research utilization. Recent advancements in RE methodologies, particularly in the context of PCKGs, have been significant. Ruan et al. (2020) demonstrated that a multi-view graph learning method could notably enhance the precision, recall, and F1 score in relation extraction from Chinese clinical records, outperforming state-of-the-art methods. Similarly, Li et al. (2019) highlighted the evolution of relationship extraction algorithms, emphasizing the role of deep learning, reinforcement learning, active learning, and transfer learning in this domain.

In the realm of therapy-disease KGs, Wang et al. (2021) constructed a Therapy-Disease KG (TDKG) using entity relationship extraction, achieving an 88.98% accuracy in extracting valid relationships from treatment- disease literature. This underscores the potential of RE in enhancing the accuracy and comprehensiveness of medical KGs. Furthermore, the integration of KGs and lexical features in biomedical relation extraction, as shown by Zhao et al. (2020), indicates significant improvements in semantic understanding. This approach exemplifies the innovative strategies adopted in recent research to enhance the effectiveness of RE in medical contexts. The development of Conditional Random Field (CRF)-powered classification models with deep learning for clinical relation extraction, as demonstrated by Lv et al. (2016), further illustrates the ongoing advancements in this field. These models have shown effectiveness in extracting clinical relation information from medical texts, thereby improving the construction of medical KGs.

In addition to NER, LLMs exhibit capabilities in processing unstructured data, enabling them to identify and extract relevant relationships between entities in clinical narratives, which is crucial for building comprehensive knowledge graphs that reflect patient information and care pathways (Liu and Fang, 2023). For instance, LLMs can automate the extraction of relationships from EHRs, thereby facilitating the integration of diverse medical data into structured formats that enhance clinical decision-making (Ge et al., 2023). Furthermore, studies have demonstrated that LLMs can achieve extraction rates as high as 87.7% in clinical contexts, outperforming some traditional NLP methods (Choi et al., 2023).

The field of NER and RE in PCKGs is rapidly evolving, incorporating cutting-edge methodologies such as multi-view graph learning, deep learning, LLMs, and knowledge-enhanced approaches. These advancements are pivotal for accurately extracting relationships from complex medical texts and enriching KGs essential for advancing patient-centered care and medical research. However, challenges like non- comprehensiveness and factfulness remain critical. Non-comprehensiveness refers to gaps where certain relevant details may be missed, especially when handling unstructured data (Nastase and Kotnis, 2019). To address this, iterative extraction techniques and the integration of multiple data sources must ensure that PCKGs provide a comprehensive view of patient information. Equally important is factfulness, as the accuracy of the extracted information directly influences patient safety and the utility of the PCKG (Radevski, 2023). Ensuring factfulness requires rigorous validation, including cross-referencing data with authoritative medical sources and employing machine learning models designed to prioritize accuracy, thereby mitigating the risks of errors in the extraction process (Lin et al., 2018).

#### 4.1.4 Knowledge representation

Knowledge Representation (KR) serves as a methodological backbone, enabling the systematic transformation of explicit and implicit knowledge into a structured and interpretable format. This transformation is crucial for machines to effectively process, analyze, and infer from the data, bridging the gap between raw data and actionable insights.

The process of KR encompasses four key components: schema definition, constraint establishment, domain knowledge encoding, and *axioms and rules utilization*, as explained in Section 3. Firstly, *schema definition* outlines entities’ structure and relationships within the KG. This step is fundamental in organizing data in a meaningful and accessible manner. Secondly, *constraint establishment* is essential for maintaining data integrity and consistency, ensuring that the relationships and entities adhere to predefined rules and norms. Thirdly, incorporating *domain knowledge* is instrumental in embedding domain-specific intelligence into the system. This allows for a nuanced understanding of the specific area of interest. Fourthly, integrating *axioms and rules* facilitates advanced reasoning capabilities, enabling the system to infer new knowledge and insights based on the existing data.

These components collectively form a robust framework that simplifies complex data and enhances the system’s capability to mirror the intricacies inherent in patient-centric data. In the following subsections, we will delve deeper into these components, exploring their roles and significance in the broader context of KR.

##### 4.1.4.1 Schema definition

The concept of *schema definition* in PCKGs revolves around structuring and organizing patient-related data to enhance understanding and interaction with this information. Schemas in KGs serve as blueprints for organizing data, enabling more effective data integration, querying, and analysis. This is particularly crucial in healthcare, where patient data is complex and multifaceted. It aims to create a unified and comprehensive view of patient information, facilitating better healthcare outcomes through informed decision-making. Ghosh and Gilboa (2014) emphasize the role of memory schemas in coordinating knowledge structures, highlighting the importance of understanding cognitive processes in schema development. This perspective is crucial in PCKGs, where patient data must be organized to align with healthcare professionals’ cognitive schemas.

Building on the concept of visual schemas by Kranjec et al. (2013), which enhance comprehension and aid in the diagnostic process, the work of Ohira et al. (2011) takes a step further by introducing the dynamic nature of graph-based data models. This flexibility is key in PCKGs, where patient data is not static but continuously evolving. The ability to dynamically add and remove relationships in these KGs ensures that they remain up- to-date and reflect the latest patient information, a critical aspect of the fast-paced healthcare environment. This dynamic nature raises an important question: should the structure of a PCKG be acyclic or allow for cycles? Acyclic graphs, such as Directed Acyclic Graphs (DAGs) (Byeon and Lee, 2023), can be beneficial in scenarios where clear, hierarchical relationships are required, particularly in representing diagnostic pathways or decision trees (Ellison, 2023). However, in many healthcare applications, patient data often involves temporal or causal relationships that inherently form cycles. For instance, a patient’s treatment may influence subsequent conditions or interventions, creating feedback loops that must be accurately represented within the graph. Therefore, allowing cycles within the PCKG may be necessary to capture healthcare data’s complex, interdependent nature, enabling a more realistic and comprehensive representation of patient health journeys (Pressat Laffouilhère et al., 2022).

Transitioning from the dynamic structuring of patient data, the focus shifts to the cognitive aspects of PCKGs, as discussed by Gilboa and Marlatte (2017). Their research underscores the significance of aligning PCKGs with the mental models of healthcare professionals. This alignment is not just about efficiently structuring data; it’s about ensuring that the KG resonates with the users’ cognitive processes, enhancing memory recall and decision-making capabilities.

Further advancing this discussion, Ji et al. (2018) delve into the intricacies of schema induction in KGs. Their exploration into the challenges and developments in this field is particularly relevant for PCKGs. As these KGs become more complex and integral to healthcare, understanding and overcoming the challenges in schema induction is paramount for ensuring that PCKGs are robust, comprehensive, and seamlessly integrated into the healthcare workflow.

##### 4.1.4.2 Constraints establishment

Establishing constraints in PCKGs focuses on enhancing the accuracy, privacy, and efficiency of medical data management and analysis. Various methodologies and strategies have been adopted to create and apply constraints in PCKGs, as evidenced by recent academic research. For example, Aguilar-Escobar et al. (2016) demonstrate the application of the Theory of Constraints (TOC) in improving the logistics of medical records in hospitals, highlighting the potential of TOC in enhancing service quality and reducing costs in healthcare settings. This approach underscores the importance of efficient data management in PCKGs.

Building on this notion of efficiency, the focus shifts to the critical aspect of privacy and security in healthcare, where Mathew and Obradovic (2011) illustrate how constraint graphs can secure privacy in medical transactions, preventing unauthorized attribute-based transformations in clinical data. This is crucial in PCKGs, where patient data sensitivity is paramount. The evolution of PCKGs further extends into dynamic data handling and adaptability. Matsuto and Shiina (1990) propose a model for medical knowledge representation based on constraints, which dynamically generates laboratory schedules using constraint propagation. This approach emphasizes the adaptability of PCKGs in accommodating patient-specific information.

Advancements in KG reasoning further enhance the adaptability of PCKGs. Wu et al. (2017) discuss a constraint-based embedding model for KG reasoning, focusing on semantic-type constraints in constructing corrupted triplets. This methodology significantly improves the reasoning accuracy of KGs, a key aspect in developing effective PCKGs. Alongside reasoning accuracy, the representation of knowledge and uncertainties also plays an important role. Pugh and Gillan (2020) introduce Propositional Constraint Graphs (PCG) to represent knowledge and uncertainties in various tasks, including healthcare. This approach aids in clearly visualizing and managing complex information in PCKGs.

Applying these concepts is not limited to static scenarios but extends to dynamic and evolving medical challenges. Tu (2021) proposes a constraint propagation approach for identifying biological pathways in COVID-19 KGs. This method uses semantically extracted information to indicate the potential for PCKGs in pandemic response and other rapidly evolving medical scenarios. Finally, integrating context-aware constraints brings a new dimension to the integrity and validation of knowledge in PCKGs. Wilcke et al. (2020) discuss using context-aware constraints to improve knowledge integrity in heterogeneous KGs. This balance of complexity and utility is crucial for knowledge validation tasks in PCKGs (Wilcke et al., 2020), highlighting constraints’ continuous evolution and application in developing robust and efficient PCKGs.

Establishing constraints in PCKGs is a multifaceted process involving the integration of various methodologies to enhance data accuracy, privacy, and efficiency. The strategies adopted in recent research reflect a growing emphasis on adaptability, security, and precision in managing patient-centric data in healthcare systems.

##### 4.1.4.3 Domain knowledge encoding

Domain Knowledge encoding focuses on integrating and representing complex medical knowledge in a structured and interconnected format. This encoding process is crucial for developing PCKGs, which enhance patient care through personalized and informed decision-making. The methodologies for creating domain knowledge encoding in PCKGs are diverse and innovative. Wang et al. (2016) proposed a framework utilizing medical chart and note data, employing a bag-of-words encoding method and a model considering both global information and local correlations between diseases. This approach underscores the importance of capturing the nuances of medical data for effective KG construction.

Further expanding on these methodologies, Ji et al. (2020) highlighted the significance of representation space, scoring function, encoding models, and auxiliary information in KG creation. This comprehensive approach indicates the multi-faceted nature of KG development, where various components contribute to the robustness and accuracy of the resulting graph. Similarly, Yuan et al. (2019) presented a biomedical domain KG construction approach that includes entity recognition, unsupervised entity and relation embedding, latent relation generation, clustering, relation refinement, and relation assignment. This method demonstrates the complexity of accurately representing medical knowledge in graph form. On the other hand, Rotmensch et al. (2017) utilized rudimentary concept extraction and three probabilistic models to construct high-quality health KGs, with the Noisy OR model yielding the best results. This study exemplifies using probabilistic models to enhance the quality of KGs. Furthermore, Yu et al. (2020) proposed a relationship extraction method for domain KG construction, obtaining upper and lower relationships from structured, semi-structured, and unstructured text. This method highlights the importance of extracting relationships from diverse data sources to enrich the KG.

These methodologies contribute to the construction of comprehensive and accurate KGs. In addition, they also pave the way for innovative applications in patient-centric healthcare.

##### 4.1.4.4 Axioms and rules utilization

PCKGs utilize axioms and rules to enhance the representation and analysis of patient data, leading to more informed healthcare decisions. Axioms in PCKGs are fundamental principles or statements accepted as accurate without proof and used to define relationships and properties within the graph. Conversely, rules are logical statements that infer new knowledge from existing data within the graph. Combining axioms and rules is essential in structuring and interpreting complex medical data, enabling more personalized and effective patient care.

The methodologies for creating axioms and rules in PCKGs vary across research works. Chu et al. (2021) demonstrated the adaptation of multi-center clinical datasets by incorporating an external KG, which enhanced patient features and improved predictions for acute kidney injury in heart failure patients. This approach signifies the importance of integrating diverse data sources and knowledge bases into PCKGs. In another study, Tomczak and Gonczarek (2012) developed a Graph-based Rules Inducer for extracting decision rules from data streams in diabetes treatment. This method supported medical interviews by tracking hidden context changes and avoiding overfitting, highlighting the importance of dynamic rule adaptation in response to evolving data. Shang et al. (2021) utilized an EHR-oriented KG system to effectively harness non-used information buried in EHRs, thereby improving healthcare quality and providing interpretable recommendations for specialist physicians.

The use of graph-based association rules for mining medical databases, as demonstrated by Alzoubi (2013), revealed the potential of discovering hidden medical knowledge, which could contribute significantly to understanding complex medical conditions like preterm birth. Similarly, RuleHub, as introduced by Ahmadi et al. (2020), is an extensible corpus of rules for public KGs, enabling users to archive and retrieve rules from popular KGs. This tool improves data understanding and reduces redundant work, illustrating the importance of rule-sharing and standardization in the field. Wright et al. (2011) developed a set of rules for inferring patient problems from clinical and billing data, which performed better than using a problem list or billing data alone. This method improved clinical decision support and care improvement, showcasing the practical application of rule-based systems in clinical settings.

Based on our review, using axioms and rules in PCKGs is a dynamic and evolving field, with methodologies ranging from integrating diverse data sources to developing specialized, disease-centric graphs. These approaches enhance the understanding of complex medical data and significantly advance personalized patient care.

### 4.2 PCKG evaluation

Evaluation of PCKGs is critical for ensuring their efficacy and accuracy in representing and inferring medical knowledge. These evaluations are typically conducted using two principal methodologies: *qualitative* and *quantitative* assessments. Qualitative assessment involves a detailed examination of the KG to ensure it aligns with clinical best practices and accurately reflects medical relationships. This method may utilize usability studies, content analysis, and expert panel reviews to gage the graph’s relevance and correctness (Rotmensch et al., 2017; Brundage et al., 2015). On the other hand, quantitative assessment employs statistical and computational techniques to measure performance metrics such as accuracy, recall, and precision, providing a numerical evaluation of the graph’s effectiveness (Rotmensch et al., 2017).

#### 4.2.1 Quantitative assessment

Quantitative methodologies for PCKG evaluation focus on numerical metrics to assess the graph’s performance. These methods include *completeness, consistency, accuracy, and embedding techniques*.

Completeness in a KG refers to the extent to which all necessary information is represented. In healthcare, this means ensuring that a PCKG includes comprehensive data on diseases, symptoms, treatments, and patient histories. A systematic literature review by Issa et al. (2021) emphasizes the importance of assessing the completeness of KGs and identifying various methodologies and metrics used for this purpose. On the other hand, consistency involves checking for logical coherence within the graph, particularly in terms of medical terminologies and relationships. This ensures that the KG does not contain contradictory information, which is crucial for clinical decision-making. The work of Sharma and Bhatt (2022) on privacy- preserving KGs in healthcare highlights the importance of maintaining consistency in data representation.

Other methods, such as accuracy assessment and embedding techniques, are integral to developing reliable PCKGs. The accuracy of a KG, as highlighted by Li et al. (2020), is crucial for patient safety and effective treatment planning, ensuring that the information aligns with authoritative medical databases and literature. Complementing this, embedding techniques, such as node2vec or GraphSAGE, as demonstrated by Talukder et al. (2022), play a vital role in transforming complex graph data into a more accessible format by capturing and representing semantic relationships. Moreover, the results of these embedding techniques can be quantitatively evaluated through tasks like link prediction, triple classification, and clustering, which provide metrics to assess the quality and effectiveness of the embeddings in representing the underlying knowledge within the PCKG.

#### 4.2.2 Qualitative assessment

Qualitative assessment methodologies are instrumental in evaluating the effectiveness of PCKGs in meeting the needs of their intended users, including clinicians, researchers, and patients.

In the qualitative assessment of PCKGs, usability studies and feedback loops play an essential role in the KG evaluation. Usability studies, as highlighted by Brundage et al. (2015), are crucial for evaluating the ease of use and accessibility of PCKGs, particularly in the context of interpreting patient-reported outcomes (PROs) in graphic format. These studies typically employ a blend of qualitative and quantitative methods, including surveys, interviews, and user testing to gather comprehensive feedback on the user experience. Complementing this, feedback loops, as discussed by Rospocher et al. (2016), enable users to provide direct input on their experiences with the KG. This feedback is instrumental in continuously refining and adapting the KG to ensure it aligns closely with user needs and preferences.

Furthermore, the quality of PCKGs is also gaged through comparison with established databases and expert validation. Benchmarking PCKGs against renowned medical databases like PubMed and ClinicalTrials.gov is essential for assessing their content coverage and accuracy (Huang et al., 2017). This comparison helps identifying any gaps or discrepancies in the PCKG. Additionally, expert validation, underscored by the work of Rotmensch et al. (2017), involves domain experts who ensure the medical relevance and accuracy of the PCKG’s content. Their insights into the clinical applicability of the information within the KG are vital for maintaining its reliability and trustworthiness.

The evaluation of PCKGs through qualitative and quantitative assessments is a multifaceted process that involves reviewing medical content and analyzing performance metrics. Progress in this field has benefitted medical professionals and patients by ensuring that PCKGs serve as reliable and effective tools.

### 4.3 PCKG processing

By processing PCKGs, healthcare providers can extract actionable insights that inform clinical decisions. The utilization of PCKGs involves different methods, including reasoning, semantic search, and inference.

#### 4.3.1 Reasoning

KG Reasoning in healthcare extends beyond mere data aggregation, playing an essential role in deriving new insights and facilitating informed decision-making. This reasoning process involves structuring information, extracting features and relations, and performing logical deductions to uncover new knowledge from existing data within the graph (Rajabi and Kafaie, 2022). For instance, in mental healthcare, KGs have been utilized for emotion recognition from facial expressions and heart rate, demonstrating the potential of knowledge reasoning in predicting emotional states (Wenying et al., 2020). Moreover, KGs in healthcare support clinical decision- making and enhance hospital efficiency by integrating heterogeneous medical knowledge and services, thereby enabling cognitive computing and semantic reasoning (Ding et al., 2021).

Furthermore, integrating LLMs with KGs can significantly improve patient engagement by empowering patients to navigate the healthcare system more effectively. With their NLP capabilities, LLMs facilitate patient-provider communication by allowing patients to ask questions and receive personalized responses tailored to their health concerns (Kassner et al., 2023). This patient-centric approach enhances the patient experience and fosters a collaborative environment where patients are more actively involved in their care decisions (Hou et al., 2023).

#### 4.3.2 Semantic search

It enables the retrieval of specific information by structuring searches to navigate the complex relationships within the graph. Various strategies have been adopted to enhance the precision and efficiency of information retrieval in PCKGs. For instance, using Semantic Web and Knowledge Management approaches has been pivotal in implementing patient-centric strategies with well-defined semantics (Lytras et al., 2009). Graph-based methods incorporating semantic-rich knowledge bases and lazy learning algorithms have shown promise in linking multimodal clinical data for improved diagnosis performance (Wang et al., 2018). Moreover, the retrieval of similar clinical cases has been refined by mapping text to Unified Medical Language System (UMLS) concepts and representing patient records as semantic graphs, demonstrating superiority over traditional models (Plaza and Díaz, 2010).

Traditional semantic search methods often rely on structured queries and entity-centric KGs, focusing primarily on discrete entities and their relationships. However, these approaches can be limited in capturing the complexities of patient data, which often includes temporal and contextual information. Recent advancements in knowledge graph technology, particularly in integrating event-centric data, have begun to address these limitations. For instance, highlight the importance of event-centric knowledge graphs, which can provide richer contextual information often missing in traditional entity-centric models (Gottschalk and Demidova, 2018). Similarly, it emphasizes that existing knowledge graphs frequently overlook temporal relationships, which is crucial for understanding patient histories and treatment timelines (Costa et al., 2020).

Integrating LLMs, such as GPT-3, into the semantic search framework of PCKGs presents a transformative opportunity to advance search capabilities beyond conventional methods. LLMs excel in understanding and generating human-like text, making them powerful tools for interpreting complex queries and retrieving relevant information from knowledge graphs. The combination of LLMs with PCKGs not only enhances the interpretability and contextual relevance of search results but also personalizes the information retrieved to align with patient-specific contexts, significantly improving the quality of healthcare decisions (Wilhelm et al., 2023). This advancement is particularly critical in healthcare, where the subtleties of patient data can greatly influence treatment outcomes. Furthermore, LLMs hold promise in supporting mental healthcare by providing contextually relevant information while ensuring clinicians remain central to the decision-making process (Torous and Blease, 2023).

#### 4.3.3 Inference

Inference uses the graph’s inherent structure to deduce new insights, facilitating the discovery of patterns and trends that may not be immediately apparent (Wang et al., 2018; Lytras et al., 2009). One prominent method for inference in KGs is using ontology-based reasoning. For instance, they demonstrated the effectiveness of ontology-based inference by utilizing the Elk reasoner to derive new triples from an existing knowledge graph, significantly expanding its informational scope (Alshahrani et al., 2017). This approach is particularly relevant in healthcare, where medical ontologies can encapsulate complex relationships among diseases, symptoms, and treatments. The ability to infer new knowledge from existing data allows healthcare providers to uncover insights that may not be immediately apparent from raw data alone. Moreover, the integration of statistical relational learning (SRL) techniques into knowledge graphs has been shown to enhance inference capabilities and reviewed various SRL techniques that can be applied to large-scale knowledge graphs, highlighting their potential to improve the accuracy and completeness of inferences made from these graphs (Nickel et al., 2016). This is particularly important in PCKGs, where the relationships between patient data points can be intricate and multifaceted. By leveraging SRL, PCKGs can provide more robust predictions and recommendations based on the underlying data. In addition to traditional reasoning methods, machine learning approaches, particularly those involving neural networks, have gained traction in KG inference. For example, recent advancements in link prediction algorithms based on neural embeddings have shown promise in completing sparse KGs (Agibetov and Samwald, 2020). These algorithms can identify missing relationships between entities in a PCKG, enhancing the graph’s utility for clinical applications. Furthermore, the work emphasizes the importance of effectively learning over multiple graphs to construct a unified representation, which is crucial for comprehensive patient-centric applications (Trivedi et al., 2018). Fuzzy inference systems (FIS) have also emerged as a valuable technique in PCKGs, particularly for diagnosing diseases. As highlighted, FIS can facilitate accurate and early diagnosis by utilizing fuzzy logic to interpret patient data (Chuan et al., 2022). This method is particularly beneficial in healthcare settings where uncertainty and variability in patient data are prevalent. By incorporating fuzzy inference into PCKGs, healthcare providers can better understand patient conditions and tailor interventions accordingly.

These methods underscore the advances in accuracy and efficiency in the processing and utilization of PCKGs, leading to enhanced healthcare outcomes.

After examining the methodologies for building, evaluating, and processing PCKGs, we move on to practical applications and use cases to demonstrate how PCKGs can be applied in real-world scenarios, showing their impact and significance in healthcare.

## 5 Applications and use cases

As the healthcare industry increasingly embraces data-driven decision-making, PCKGs have emerged as a powerful tool for personalized medicine. These KGs, which place the patient at the center of a complex network of interconnected health data, offer a holistic view of a patient’s health journey. They encompass various data, from medical history and genetic information to lifestyle factors and environmental influences. The applications of these KGs are broad and innovative, offering the potential for improved disease prediction, enhanced treatment planning, and more effective preventive strategies. In the following subsections, we will explore these applications in detail, shedding light on how PCKGs revolutionize healthcare and pave the way for a more personalized, predictive, and proactive approach to patient care.

### 5.1 Predicting disease before onset

The healthcare sector has seen significant advancements in patient-centric solutions by integrating KGs. Using a KG to proactively forecast the likelihood of a disease developing in an individual before any symptoms appear involves synthesizing various data points related to patient history, genetic information, lifestyle factors, and broader medical knowledge to identify patterns and risk factors associated with diseases. This predictive model allows for early intervention strategies, significantly altering the disease trajectory and improving the patient’s long-term health outcomes.

The methodologies for creating PCKGs for disease prediction are diverse and innovative. Chen et al. (2019) discuss the robust extraction of medical knowledge from EHRs to build graphs that evaluate accuracy across different diseases and patient demographics using non-linear functions for causal relationships. Similarly, Talukder et al. (2022) describe an AI-based approach integrating disease-related knowledge bodies with Node2VEC for link prediction in disease-symptom networks. Furthermore, Liang et al. (2022) highlight using multi-hop reasoning over KGs, which provides interpretability and is superior to single-hop methods. Similarly, refining of medical KGs using latent representations aids in prediction and maintains the explainability of diagnoses (Heilig et al., 2022). Expanding on these advancements, Li et al. (2020) introduce a Graph Neural Network-Based Diagnosis Prediction (GNDP) model that uses spatial–temporal graph convolutional networks for diagnosis predictions. In a complementary manner, Zheng et al. (2021) proposed a Multi-modal Graph Learning framework (MMGL) that exploits multi-modality information for disease prediction.

Different strategies across research works include the use of random walk along KG (Sun et al., 2020), Graph Neural Networks (GNN) for embedding medical concepts (Sun et al., 2020), and hybrid systems combining KGs with clinical experience for pediatric disease prediction (Liu et al., 2017). Zhang (2021) built an automatic question-answering system based on medical KGs, while Saha et al. (2020) predicted missing and noisy links in clinical KGs using neighborhood-based embeddings. Key findings from our literature review indicate significant advances in disease prediction accuracy, efficiency, and outcomes. For instance, the integration of GNNs with EMR data has led to highly representative node embeddings that improve prediction accuracy (Sun et al., 2020).

The use of tensor factorization on biological KGs for predicting co-morbid disease pairs (Biswas et al., 2019) and the construction of KGs based on evidence-based medicine for diabetes complications (Wang et al., 2020) is a notable advance.

PCKGs represent a significant step forward in the field of disease prediction. They offer the potential to transform patient outcomes by enabling early detection and personalized treatment plans. Future implications include the continued refinement of these models to enhance their predictive power and the integration of even more diverse data sources to capture the full spectrum of patient health and disease progression. In Table 2 we summarize a list of selected disease prediction applications in terms of their impact and limitations in the field of PCKGs.

**Table 2:** A summary of selected literature on PCKGs for “*Predicting Disease Before Onset*.”

Paper title	Focus/objective	Contribution(s)	Limitation(s)
**Predicting disease before onset**			
A novel link prediction approach on clinical knowledge graphs utilizing graph structures (Dörpinghaus et al., 2022)	The goal is to create a proof of concept showcasing the efficacy of graph structures in AI methodologies	Development of a graph-based method merging Conditional Random Fields (CRFs) and graph embedding for knowledge discovery This method successfully predicts labels for graph nodes with high precision and recall	The method is time-intensive when querying features from the graph, particularly with large datasets Increased runtime and memory demands for multi-node paths in the graph
Deep knowledge reasoning guided disease prediction (Chuan et al., 2022)	The paper aims to enhance model interpretability by integrating knowledge entities with single-hop and multi-hop relationships	Introduction of HitaNet, a hierarchical time-aware attention network HitaNet uses a self-attention based transformer model for enhanced disease prediction The paper presents a unique token wrapping method to merge knowledge graph insights with EHR data	Lower time efficiency compared to baseline methods as a limitation The proposed method’s effectiveness diminishes with the availability of sufficient data
Predicting missing and noisy links via neighborhood preserving graph embeddings in a clinical knowledgebase (Saha et al., 2020)	The paper proposes a model that combines support vector classification (SVC) and neural network-based probabilistic embedding (NPE) to predict the links between clinical entities in the KG	The development of a novel approach that integrates SVC and NPE for link prediction in KGs The model achieved promising results in terms of predicting missing associations	The reliance on existing clinical databases, which may contain noisy or incomplete data The potential mismatch between model predictions and clinical recommendations
Relation prediction of co-morbid diseases using knowledge graph completion (Biswas et al., 2019)	The objective is to predict relationships between co-morbid diseases using knowledge graph completion techniques	Proposing a method that combines distributed representation learning and graph embedding to predict disease-disease relationships Development of a novel approach that leverages the structure and semantics of a KG to predict disease relationships	The proposed method relies on the availability of a comprehensive and accurate knowledge graph The evaluation of the method is limited to a specific dataset
Disease prediction via graph neural networks (Sun et al., 2020)	The paper addresses the challenges in predicting both common and rare diseases by integrating expert knowledge with machine learning techniques	Proposing a systematic solution that combines expert knowledge with machine learning Introducing a novel graph embedding-based model for disease prediction, which learns embeddings from medical concept graphs and patient record graphs	Struggling to adapt to rare diseases due to data scarcity and complex symptom-diagnosis relationships Reliance on historical patient records for model training limits the ability to serve new patients

### 5.2 Recommending individualized interventions

Intervention recommendation aims to use KGs to suggest tailored medications and treatments. By mapping patients’ unique health profiles, these graphs enable physicians to pinpoint optimal therapies that enhance the precision of medical decisions, leading to improved patient outcomes. The application of KGs in personalized treatments is predicated on integrating diverse data sources, including EHRs, clinical notes, and patient-generated data, to construct a comprehensive, interconnected data structure that reflects individual patient profiles. This patient-centric approach is crucial as it allows for treatments to be tailored based on each patient’s unique medical history, genetic information, lifestyle, and preferences, which is a departure from the one-size-fits-all healthcare model.

The approaches to developing and implementing PCKGs differ among research studies, but they all aim to achieve robustness, accuracy, and personalization. For instance, Gyrard et al. (2018) aggregate knowledge from IoT devices, clinical notes, and EMRs to manage chronic diseases, showcasing the integration of AI and machine learning in constructing Personalized Healthcare Knowledge Graphs (PHKGs). Transitioning from a general approach to a more specialized application, Individualized Knowledge Graphs (IKGs) in cardiovascular medicine are one strategy for developing these KGs, which combine biological knowledge with medical histories and health outcomes to create personalized treatment strategies (Ping et al., 2017). Further refining the methodological framework, Rotmensch et al. (2017) utilized concept extraction and probabilistic models, finding the Noisy OR model particularly effective for constructing high-quality health KGs. Building on these methodologies, Shirai et al. (2021) applied Personal Knowledge Graphs (PKGs) to integrate patient-specific information into decision-making tools for personalized healthcare. In a similar vein of enhancing disease treatment strategies through personalized data, Zhu et al. (2022) introduced a KG that enhances rare disease (RD) treatment recommendations by systematically compiling and semantically annotating RD-related scientific articles, aggregating essential research findings and therapeutic insights with a sophisticated data model.

Literature has demonstrated significant advances in treatment accuracy, efficiency, and outcomes. For instance, the Four-Tuple Path Matrix in Traditional Chinese Medicine has been proposed to create personalized KGs, enhancing diagnostic modalities (Xie et al., 2018). Li et al. (2014) demonstrated how personal KGs could be automatically constructed from user utterances in conversational dialogs, indicating the potential for real-time, dynamic treatment adjustments. PCKGs provide multiple advantages. By analyzing patient profiles using KGs, treatments are more accurate and efficient than traditional methods. For example, Zhang et al. (2022) developed an intuitive graph representation of knowledge for nonpharmacological treatment of psychotic symptoms in dementia, potentially transforming care strategies for such complex conditions.

There are many potential implications for future personalized treatment and patient outcomes. A more informed and dynamic approach to treatment has the potential to enhance medical precision, improve patient engagement and optimize health outcomes by integrating PCKGs. These methods are expected to become more sophisticated as the field evolves, allowing for even greater personalization and efficacy in treatment. In Table 3, we summarize a list of selected treatment decision applications in terms of their impact and limitations in the field of PCKGs.

**Table 3:** A summary of selected literature on PCKGs for “*Recommending Individualized Interventions*.”

**Paper title**	**Focus/objective**	**Contribution(s)**	**Limitation(s)**
**Recommending individualized interventions**			
Individualized knowledge graph (Ping et al., 2017)	Envisioning individualized Knowledge Graphs (iKGs) in cardiovascular medicine Proposing a modern informatics platform for transforming clinical and scientific discovery	Introducing the concept of iKGs for aggregating and presenting individualized cardiovascular health data Highlighting the role of iKGs in linking biological and clinical knowledge of individual patients	Acknowledging challenges in data fragmentation, noncommensurability, and semantic inference within cardiovascular data
Personalized health knowledge graph (Gyrard et al., 2018)	Aims to manage chronic diseases more effectively using IoT data analytics and explicit knowledge	Proposes a methodology to build PHKG, integrating heterogeneous data sources Offers a solution for contextualizingand personalizing healthcare information	The paper acknowledges the complexity in semantic integration of diverse data It highlights the challenges in tailoring generic knowledge to individual patients
Developing an intuitive graph representation of knowledge for nonpharmacological treatment of psychotic symptoms in dementia (Zhang et al., 2022)	Develop a knowledge graph for nonpharmacological treatment of psychotic symptoms in dementia Enhance understanding and management of dementia-related psychotic symptoms through nonpharmacological methods	Creation of the Dementia-Related Psychotic Symptom Nonpharmacological Treatment Ontology (DRPSNPTO) Improvement in visualization and computerization of gerontological knowledge	
Learning a health knowledge graph from electronic medical records (Finlayson et al., 2014)	Automatically learn a health KG from EMRs to link diseases and symptoms and improve clinical decision-support systems	A methodology for deriving health KG from EMR using probabilistic models Demonstration that the noisy OR model significantly outperforms other tested models	Inherent difficulties in interpreting EMR data, especially the presence of complex patient conditions The reliance on rudimentary concept extraction pipelines Limitations related to the automatic inference of causal relationships from observational data
Applying personal knowledge graphs to health (Shirai et al., 2021)	The paper focuses on leveraging PHKGs to enhance healthcare decision-making by integrating personal health information with broader knowledge graphs	Proposing a conceptual framework for PHKGs, highlighting how they can support personalized, knowledge-driven healthcare applications by leveraging data from EHRs, IoT devices, and other health-related data sources	Collecting and storing personal health knowledge from heterogeneous sources Linking personal health knowledge to external KGs enhances the PHKG with broader contextual information Maintaining the PHKG to ensure it remains up-to-date and accurate

### 5.3 Enhancing clinical trials

KGs represent a paradigm shift in clinical trial patient selection by offering a structured, interconnected data framework that can encapsulate complex patient information, medical histories, and potential trial criteria. In clinical trials, patient-centric approaches are crucial because personalized medicine tailors treatments to each patient’s characteristics, necessitating a comprehensive understanding of patient data.

PCKGs are created and applied using diverse methodologies. Gortzis and Nikiforidis (2008) described an N-tier system that combines KGs with human collaboration and scalable knowledge engineering tactics. Expert input must be combined with scalable data structures to select patients effectively. Xiang et al. (2019) highlighted the standardization and structural integration provided by KGs, essential for auxiliary diagnosis systems in clinical trials. Strategies for developing KGs for patient selection in clinical trials include linking of multimodal data types for automatic diagnosis (Wang et al., 2018). Nicholson and Greene (2020) discussed machine learning methods for constructing low-dimensional representations of KGs, which support applications in genomics, pharmaceutical, and clinical domains.

Various studies have indicated significant improvements in the accuracy, efficiency, and outcomes of clinical trial patient selection. For instance, the Safe Medicine Recommendation (SMR) framework by Wang et al. (2017) bridges electronic medical records with medical KGs to learn patient-disease-drug embeddings, enhancing the precision of clinical trial patient selection. Compared with traditional ways of selecting patients for clinical trials, PCKGs offer a more thorough and subtle approach. Several studies have demonstrated the effectiveness of KGs in improving clinical trial design and outcomes by providing a holistic view of patient data, facilitating personalized trial matching, and providing a more holistic view of patient data (Sharma, 2015; Weng et al., 2017; Huang et al., 2017). Ultimately, integrating patient-centric KGs in selecting clinical trial participants can transform the field by improving the precision and personalization of patient care. As a result of a better understanding of patient data, future trials will likely be more adaptive, patients will be more engaged, and outcomes may be improved. In Table 4, we summarize a list of selected treatment decision applications in terms of their impact and limitations in the field of PCKGs.

**Table 4:** A summary of selected literature on PCKGs for “*Enhancing Clinical Trials*.”

Paper title	Focus/objective	Contribution(s)	Limitation(s)
**Recommending Individualized Interventions**			
Knowledge graph-based clinical decision support system reasoning (Xiang et al., 2019)	The focus of the paper is to highlight the benefits of using knowledge graphs over traditional hand-crafted rule databases in CDSSs	The introduction of the Path-Ranking Algorithm (PRA) as a method for automatically discovering symptoms without human intervention	The probability of certain paths in the KG may not be accurate The lack of details on the classification model used
Constructing knowledge graphs and their biomedical applications (Nicholson and Greene, 2020)	Examining the construction and application of biomedical knowledge graphs Emphasizing how machine learning is transforming these processes	Discussion of knowledge graph construction, including manual curation and text mining Review of representational learning techniques and their applications in biomedical fields	Need for advanced techniques to handle complex sentence structures Limitations in current methods to represent diverse relationships in KGs Scalability and memory limitations in matrix factorization techniques
SMR: Medical knowledge graph embedding for safe medicine recommendation (Sharma, 2015)	Developing a framework to recommend safe medicines by leveraging a heterogeneous graph that integrates patient data, diseases, and medicines	Development of graph-based embedding models enabling the recommendation of newly emerged medicines effectively A novel method to recommend safe medicines for new patients and minimizing potential adverse drug reactions Introduction of the SMR framework as a new approach to the link prediction problem	Dealing with the challenge of recommending safe medicines, especially new ones, to patients Minimizing potential adverse drug reactions in medicine recommendations is critical to patient safety
Patient centric approach for clinical trials: Current trend and new opportunities (Weng et al., 2017)	Exploring the shifting paradigm in clinical trials toward a more patient-centric model	Identifying new opportunities for the clinical research industry to adopt patient-centric approaches to accelerate drug development and improve trial outcomes	The complexity and rising costs of clinical research Ensuring data transparency and building trust with patients participating in clinical trials
Automatic diagnosis with efficient medical case searching based on evolving graphs (Lytras et al., 2009)	Developing a method for automatic diagnosis by improving medical case searching using evolving graphs, which dynamically incorporate new medical cases and knowledge	Introduction of an evolving graph framework that integrates new medical cases and knowledge A novel method for medical case searching that leverages the evolving graph structure An optimization strategy for embedding learning in the heterogeneous graph	Handling the dynamic nature of medical knowledge and cases Balancing the computational complexity of embedding learning in a continuously evolving graph structure Scalability and maintaining high accuracy and efficiency as the graph expands

Although PCKGs have proven valuable in recommending individual interventions, predicting disease before onset, and improving clinical trials, their utility goes far beyond these. Using KGs in other innovative healthcare applications is increasingly becoming common, such as optimizing hospital workflows, tailoring patient engagement strategies, and even developing telemedicine platforms. With these innovative tools, healthcare can be revolutionized by providing a more holistic, integrated view of patient data and new opportunities for research and treatment methods. Having explored the diverse applications and use cases of PCKGs in various domains, we now focus on this field’s challenges and future directions. This transition allows us to critically examine the current limitations and envision potential advancements that could further enhance the utility and effectiveness of PCKGs.

## 6 Research challenges and discussion

PCKGs in healthcare are designed to provide a comprehensive, unified view of patient data by integrating information from various sources, including EHRs, medical literature, and patient-generated data. These graphs aim to support better clinical decision-making and personalized patient care by representing complex medical data in an interconnected format that is more accessible and actionable for healthcare providers.

The current state of research in PCKGs is focused on overcoming several key challenges to maximize their potential in healthcare. Ji et al. (2020) discuss the difficulties in knowledge acquisition, completion, and temporal KG development, which are crucial for maintaining up-to-date and comprehensive patient profiles. Building on this foundation, Chen et al. (2019) highlight the need for robustness in PCKGs, particularly in addressing sample size limitations and unmeasured confounders, to extend models to larger patient visits. Moreover, Rastogi and Zaki (2020) emphasize the importance of designing, building, and operationalizing PCKGs tailored to individual patients. However, a significant hurdle remains in the actual construction of PCKGs. As noted by Gyrard et al. (2018) and Cong et al. (2018), the complexities in constructing PCKGs are often time-consuming and heavily reliant on source data quality. A significant challenge in applying traditional KG embedding methods to patient-centric healthcare applications is their struggle with structural sparsity. Hu et al. (2021) argue that conventional techniques, such as TransE and ConvE, while adept at mapping entities and relationships into a vector space, falter because they rely solely on KG triplets, neglecting the rich auxiliary texts that describe entities. This limitation significantly limits the comprehensiveness and utility of KGs in capturing detailed patient information and medical knowledge, indicating a key challenge in leveraging KGs for complex healthcare applications.

Data quality and standardization remain significant challenges, as heterogeneous data structures, poor data quality, and varying medical standards complicate data integration into a coherent KG (Zhang et al., 2020). Integrating PCKGs into clinical workflows also presents challenges, as it requires the development of systems that complement healthcare providers’ routines without causing disruptions (Gortzis and Nikiforidis, 2008). Additionally, scalability is another concern, as PKGs must be able to incorporate an ever-increasing amount of data from diverse sources, including genomic information and patient lifestyle data (Wang et al., 2018). Patient data privacy is a critical concern, particularly in utilizing PCKGs, which involve handling sensitive personal health information and require strict privacy controls to safeguard patient confidentiality. Using patients’ data for various purposes, such as consultations, research, and emergencies, poses a significant challenge for authorization systems, emphasizing the need for robust privacy protection (Al-Zubaidie et al., 2019; Sharma and Bhatt, 2022). Furthermore, real-time data analysis within PCKGs is technologically demanding, requiring advanced computational methods to process and analyze data promptly for it to be clinically relevant (Hooshafza et al., 2021).

Given these challenges, it becomes clear that continued technological and methodological innovations are necessary to enhance PCKGs’ predictive capabilities. Advanced analytics, machine learning, and semantic web technologies could be key. Additionally, as PCKGs become more integrated into healthcare delivery, addressing regulatory and ethical considerations becomes increasingly essential. This requires collaboration among computer scientists, healthcare professionals, and policymakers to align the development of PCKGs with broader healthcare objectives.

## 7 Conclusion and future directions

This literature review of PCKGs explores their development, evaluation, processing techniques, applications, challenges, and prospects. PCKGs represent a field in healthcare informatics that aims to revolutionize personalized patient care by integrating and synthesizing diverse healthcare data sources. The review highlights the current state-of-the-art methodologies for constructing and evaluating PCKGs, emphasizing the importance of qualitative and quantitative approaches to assess their effectiveness in healthcare settings. In addition to construction and evaluation, the review delves into innovative processing techniques such as reasoning, semantic search, and inference. These techniques significantly enhance the accuracy and efficiency of PCKGs, ultimately improving patient-centered care. Furthermore, exploring different applications of PCKGs in healthcare—including disease prediction, personalized treatment recommendations, and advancements in clinical trials—reveals their potential to transform healthcare through personalized and predictive medicine.

Future directions in PCKG research include leveraging advanced analytics and machine learning to improve predictive capabilities, which could lead to more accurate and timely interventions (Wu, 2021). In addition, semantic web technologies are also predicted to play a significant role in enhancing the accessibility and utility of PCKGs (Huang et al., 2017). Building on this momentum, personalized medicine emerges as a promising area where PCKGs can substantially impact. By linking genomic data with clinical outcomes, treatments can be tailored to individual patients, offering a more personalized approach to healthcare (Chandak et al., 2023). To further this advancement, methodological innovations, such as new algorithms for data harmonization and user interface design, are crucial. These innovations are needed to address current challenges and facilitate the broader adoption of PCKGs in clinical practice (Peng et al., 2023). Simultaneously, as PCKGs become more integrated into healthcare delivery, regulatory and ethical considerations gain prominence. These aspects are critical to ensuring that the deployment of PCKGs adheres to the highest standards of patient care and data management. Therefore, cross-disciplinary collaboration becomes essential for advancing PCKG technology. This involves computer scientists, healthcare professionals, and policy-makers working together to ensure that the development of PCKGs aligns with broader healthcare objectives and respects ethical guidelines (Solanki et al., 2022; Maxhelaku et al., 2022).

PCKGs represent a significant advancement in healthcare informatics. They can transform patient outcomes by enabling early detection and personalized treatment plans. Despite the challenges involved, the future outlook for PCKGs is promising, as they have the potential to improve patient care and healthcare delivery significantly. Ongoing research efforts and interdisciplinary collaboration will be crucial in fully realizing their novel impact on healthcare.
